# Impact of Hurricane Maria on mold levels in the homes of Piñones, Puerto Rico

**DOI:** 10.1007/s11869-022-01297-7

**Published:** 2022-12-26

**Authors:** B. Bolaños‑Rosero, X. Hernández‑González, H. E. Cavallín‑Calanche, F. Godoy‑Vitorino, S. Vesper

**Affiliations:** 1 Department of Microbiology and Medical Zoology, University of Puerto Rico, San Juan, PR, USA; 2 School of Architecture, University of Puerto Rico, San Juan, PR, USA; 3 Center for Environmental Measurement and Modeling, United States Environmental Protection Agency, Cincinnati, OH, USA

**Keywords:** Puerto Rico, Mold, Hurricane, Housing, Construction materials

## Abstract

Hurricane Maria struck Puerto Rico on September 20, 2017, severely impacting the island. In order to quantify the impact of the hurricane on the indoor air quality, we evaluated the fungal levels in households (*n* = 20) of the Piñones community for the period of 2018 and 2019. For each dust sample collected, the 36 Environmental Relative Moldiness Index (ERMI) molds were quantified using qPCR assays, and then Shannon Diversity Index (SDI) values for the fungal populations were calculated. Homes were in five separate regions, regarding their proximity in the studied area. We found that for regions with reported least water damage, the SDI values were similar for both sampled years, but for regions that reported mid-to-high level of damage region, the SDI values were significantly higher. Households that reported remediation actions between the two sampled years showed similar values for the second year as those that did not report any major impact. Our preliminary data provides insights into the significant impacts of hurricanes into indoor fungal environment.

## Introduction

On the morning of Wednesday, September 20, 2017, Hurricane Maria struck Puerto Rico with a category 4 wind force and heavy rains. Much of the island was without power for months. The hurricane had devastating effects on the environment and people. These environmental impacts included destruction of the island’s vegetation (Scholl et al. 2021) and housing infrastructure (Ma and Smith 2020). Renters and low-income families were found to be most vulnerable to Maria’s impact (Ma and Smith 2020). With climate change, hurricanes are expected to increase in number and intensity with impacts on the environment and diseases like asthma (D’Amato et al. 2020; El-Sayed and Kamel 2020).

Even before the hurricane, Puerto Rico had high rates of asthma, approximately 16.5% compared to 8.4% for mainly the United States (US) (US CDC 2012). In 2000, 30.6% of interviewed subjects reported having one to three visits to emergency departments as a result of asthma in the previous year (Pérez-Perdomo et al. 2003). In some areas of Puerto Rico, 46% of elementary children had asthma (Loyo-Berríos et al. 2006).

A study of healthcare utilization in Puerto Rico in 2013 demonstrated that increases in mold and PM_10_ were associated with increases in asthma claims (Lewis et al. 2020). In 2014–2016, an estimated 53% of adults with asthma and 29% of children with asthma in Puerto Rico had uncontrolled asthma (Cowan et al. 2020). Adults and children with uncontrolled asthma were significantly more likely to have observed mold in their homes than were those with controlled asthma. Blatter et al. (2014) suggested that indoor fungal exposure leads to an increased degree of atopy and visits to the emergency departments or urgent care facilities for asthma in Puerto Rican children.

Exposures to damp-indoor, moldy environments are linked to asthma based on many scientific reviews (IOM 2004; WHO 2009; Quansah et al. 2012; Sharpe et al. 2015). Therefore, it is important to have quantitative methods to describe mold contamination levels in homes. The US Environmental Protection Agency in conjunction with the Department of Housing and Urban Development created the Environmental Relative Moldiness Index (ERMI) scale to standardize the assessment of the mold contamination in US homes (Vesper et al. 2007).

The ERMI metric is based on the analysis of 36 widely distributed indicator molds: 26 group 1 molds, which are associated with water damage in homes, and 10 group 2 molds, which primarily enter the home from the outside environment (Vesper 2011). The ERMI value for a home is calculated, as shown in Eq. 1. The summed common logs of the concentrations of the group 2 molds (*s*_2_) were subtracted from the summed common logs of the concentrations of group 1 molds (*s*_1_) to produce the ERMI value (Vesper et al. 2007). This metric was

(1) $$\text{ ERMI}=\sum_{i=1}^{26}\log_{10}(s1i)-\sum_{j=1}^{10}\log_{10}\left(s2j\right)$$

previously applied in a pilot study of molds in settled dust from inside eastern Puerto Rican homes (Bolaños-Rosero et al. 2013).

The objective of this study was to determine the impact of Hurricane Maria on fungal levels in homes located in the low-income community (69.8% living below the federal poverty level) of Piñones located in the Loíza municipality on the northeastern coast of Puerto Rico. The residents (about 2500) are predominantly black Hispanic with 35.8% of the population under 18 years of age in 2010, the latest available data (US Census 2010). Three natural barriers (a river, a large mangrove forest, and the ocean) separate Piñones from the rest of the municipality, leading to greater environmental exposures and reduced healthcare access (closest clinic is about 15 km away) (García-Rivera et al. 2017). Understanding the impact of events like hurricanes on this environmental justice community is necessary to correct housing disparities and increase resiliency to natural disasters.

## Material and methods

### Home selection

**Fig. 1:**
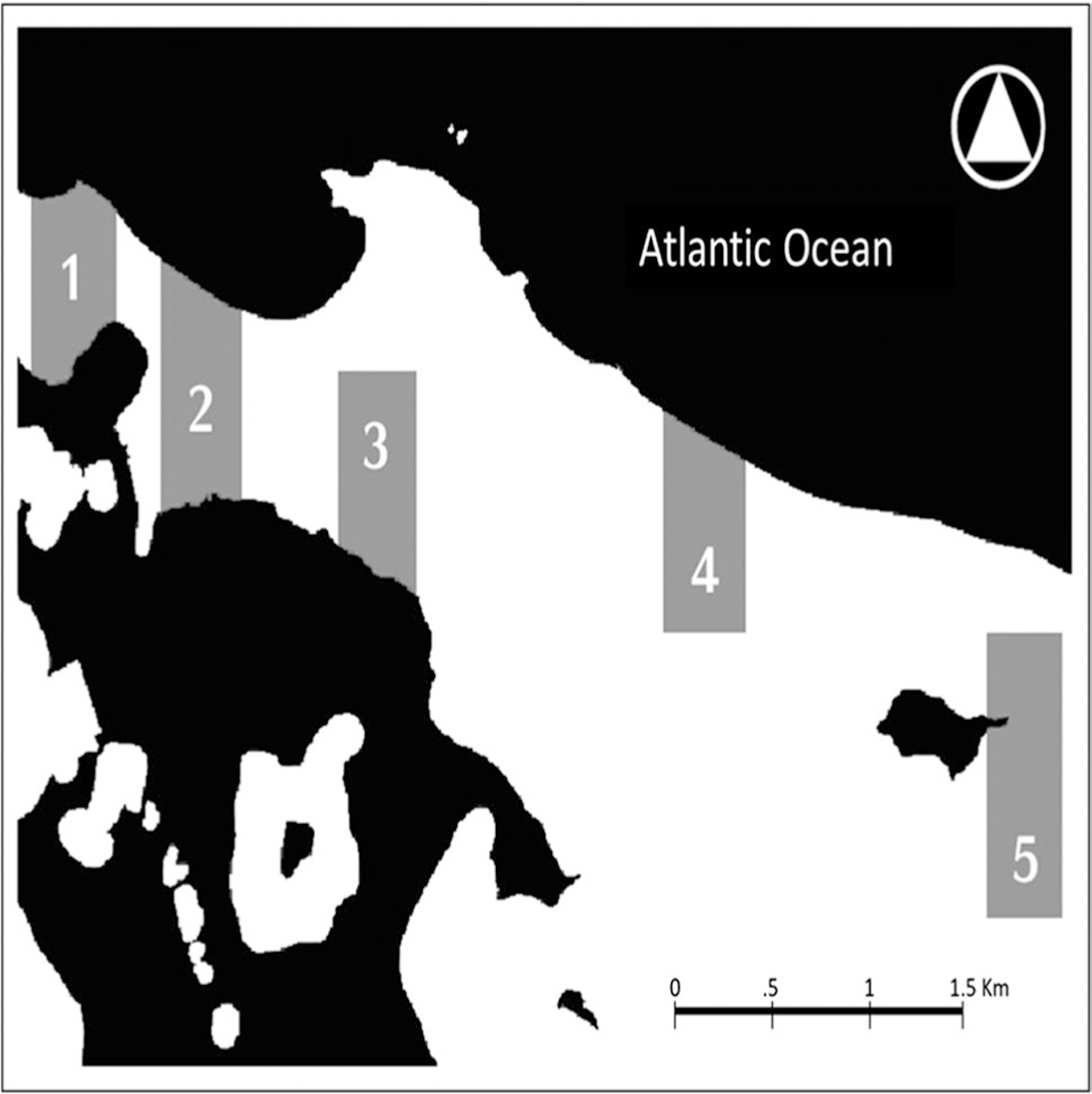
Sampling locations in regions 1 to 5 in Piñones. The gray areas of the map show the footprint of the regions in Piñones community and each region’s proximity to water, shown as the black areas

Because of the sensitive conditions after the event, participants in this research were selected by means of their interest and their availability to participate in the study. The volunteered participant’ households were categorized based on their proximity to the local bodies of water, by considering that the exposure to them could be a factor affecting both the initial impact and then the long-term effect in the sampled households. Based on this, we divided that sampled households into five different regions (region 1, *n* = 6; region 2, *n* = 5; region 3, *n* = 2; region 4, *n* = 5; and region 5, *n* = 2) within the community of Piñones (Fig. 1). This study is cross-temporal, taking measurements for both years 2018 and 2019. For the second year (2019), some families declined to continue as part of the study.

Because of IRB requirements, participants in the study were selected by their interest to participate. In a post hoc evaluation of the participant household locations, we decided to group these households based on their proximity regarding location. Households for each of the five regions of analysis are located in proximity to each other by less than 100 m, and each of these regions is separated by 300 m or more. The assumption for the post hoc grouping was that proximity/separation of households to each other could impact potential similarities/differences for the studied households.

This study was approved by the IRB Committee of the Medical Sciences Campus, UPR (A9830117) and the CIPSHI of the University of Puerto Rico-Rio Piedras (1617–033).

### Home assessments

The studied area was subject to the impact of both Hurricanes Irma and Maria during 2017. The area was not reported as flooded, but households were subject to the impact of rain and winds, and households suffered damaged ranking from minor (water entering through windows) to major (structural damage to roof and/or walls).

In order to document the conditions of the households, we identified the owner of the participant homes, and we assed via interviewing basic information regarding exposure to damage, repairs to the households after the event, material characteristics of the households, type of ventilation of the spaces, and presence of pets (see Table 1). These variables were collected as literature has shown potential connections between them and the occurrence of fungi in households.

### Dust sampling

Dust samples were collected in different areas within the living room and bedroom, (e.g., door frames, bookshelves, and windowsills), using a clean electrostatic cloth (EC) (Swiffer^™^, Proctor and Gamble, Cincinnati, OH). Once the cloths were completely impregnated with dust (maybe describe here what means “completely impregnated with dust”); it was placed in sealable plastic bags (Ziploc^®^, SC Johnson, Racine, WI). The cloths that contained the dust samples were placed in a cooler (4 °C) and returned to the laboratory where they were stored at − 80 °C until analyzed.

### Quantitative PCR (qPCR) analyses of dust samples

The EC dust was recovered, as previously described (Cox et al. 2017). Five milligrams of sieved (pore 300 μm) dust from each sample were added to a 2-ml extraction tube containing 0.3 g of glass beads, as previously described (Haugland et al. 2004). Each EC or vacuum dust sample was spiked with 1 × 106 conidia of *Geotrichum candidum* at the time of extraction as an internal reference to ensure that the extraction and purification were performed correctly (Haugland et al. 2004). A bead beater (BioSpec Products, Bartlesville, OK) was used to shake each extraction tube at 5000 rpm for 1 min to release the DNA from the cells. The DNA was then purified using the DNA-EZ extraction kit (GeneRite, Monmouth Junction, NJ), following the manufacturer’s instructions.

Each of the 36 ERMI molds was quantified in each extract with qPCR assays (Haugland et al. 2002). The standard qPCR assay contained 1 μl of a mixture of forward and reverse primers at 25 μM each, 12.5 μl of “Universal Master Mix” (Applied Biosystems Inc., Foster City, CA), 2.5 μl of 2 mg ml^−1^ fraction V bovine serum albumin (Sigma Chemical, St. Louis, MO), 2.5 μl of a 400 nM TaqMan probe (Applied Biosystems Inc., Foster City, CA), and 2.5 μl of DNA-free water (Cepheid, Sunnyvale, CA). Five microliters of the DNA extract from the sample and this mix were combined. Reactions were performed with thermal cycling conditions consisting of 2 min at 50 °C, 10 min at 95 °C, followed by 40 cycles of 15 s at 95 °C for template denaturation, and 1 min at 60 °C for probe and primer annealing and primer extension, and 5 mg from each of the sieved-dust samples was analyzed by a commercial laboratory that performs the ERMI analysis (Mycometrics LLC, Monmouth Junction, NJ).

### Analyses

The average concentration of each of the 36-ERMI molds in the dust samples from 2018 to 2019 were compared using the Wilcoxon rank sum test, corrected for multiple comparisons using the Holm–Bonferroni test. The average sum of the logs of the group 1 molds, sum of the logs of the group 2 molds, or ERMI values were compared between sampling years using the Student’s *t*-test (Table 2).

The diversity of mold populations in the homes in the study was determined with the Shannon index of equitability (Shannon & Weaver 1963). The diversity boxplots were built using R’s ggplot2 package (Wickham 2009) from a species table and two corresponding files, taxonomy and metadata, as a phyloseq object, and alpha diversity was plotted with the “plot_richness” function (McMurdie & Holmes 2013) using year and region as sample groupings. Statistical tests for SDI values by year and region were done using libraries “ggpubr” and “ggplot2” in R (Kassambara 2018) by calculating pairwise differences between region level means with Tukey’s multiple comparisons of means tests.

## Results

Our project calculated the comparisons between the average population of each of the 36-ERMI molds in the homes in the community of Piñones in 2018 and 2019. Neither the sum of the logs of the group 1 molds nor the ERMI values changed significantly between 2018 and 2019. However, the sum of the logs of the group 2 molds did increase significantly in 2019 compared to 2018 (7.8 vs 4.8, respectively) (Table 2). There were two species of group 2 molds, *Alternaria alternata* (*p* < 0.001) and *Cladosporium cladosporioides* Type 1 (*p* < 0.001), whose concentrations indoors were significantly greater in 2019 than 2018 (Table 2).

**Fig. 2:**
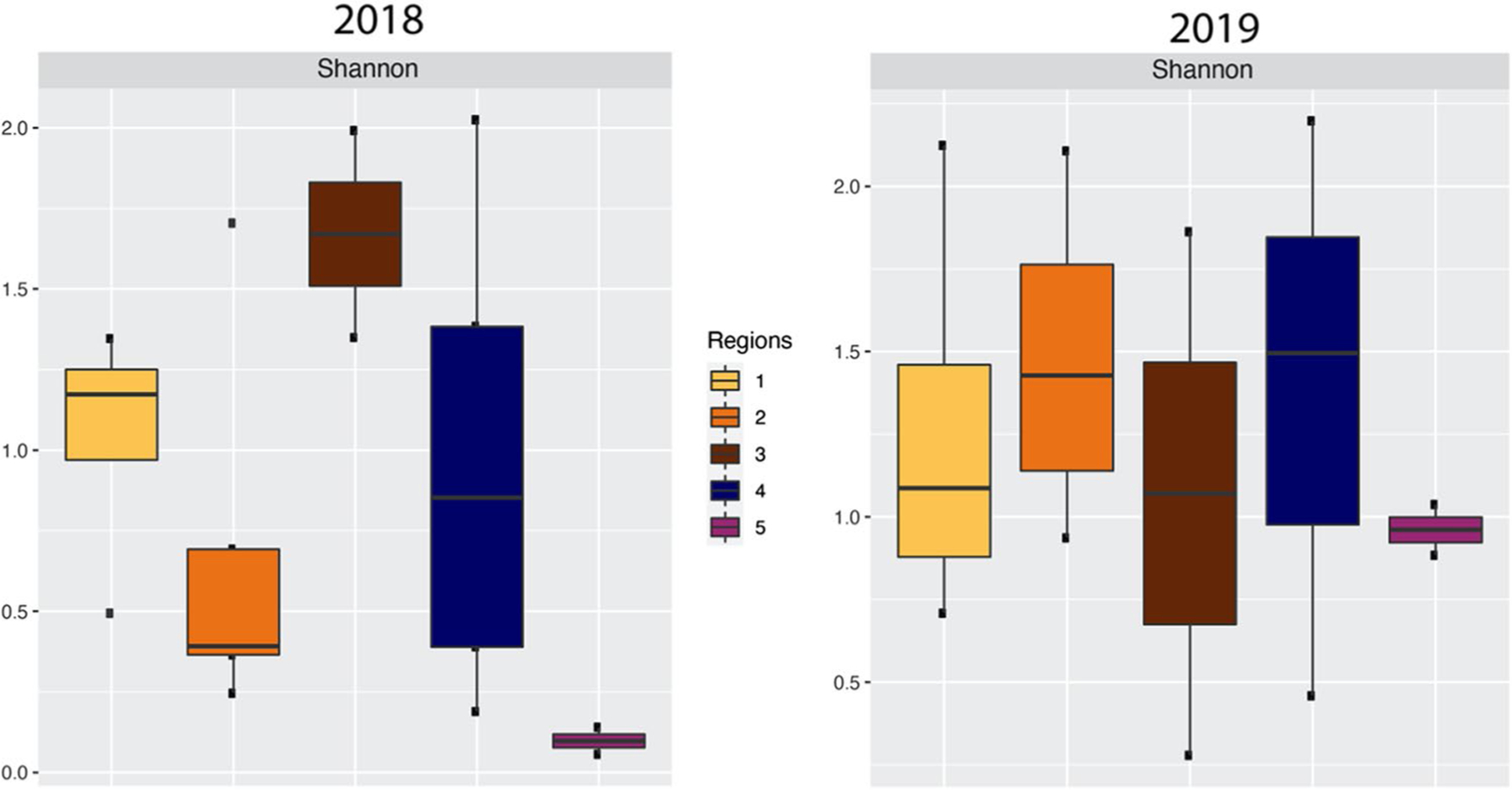
Shannon Diversity Index (SDI) values for the five regions in Piñones, in 2018 and 2019. Tukey HSD (honestly significant difference) tests were applied to the mean values of each region; none of the pairwise comparisons were significantly different after *p*-adjustment (see suppl table 1)

Next, the change in the diversity of the mold populations in the homes in each of the regions were compared between 2018 and 2019 by plotting the Shannon Diversity Index (SDI) value (Fig. 2). The homes in regions 1 and 4 were spared of water damage from Hurricane Maria. Two of the homes in region 2 received some water damage. The SDI values for homes in regions 1, 2, and 4 changed a little between 2018 and 2019 (Fig. 2). In region 3, the SDI value was the highest of any region in 2018 and only decreased slightly in 2019. In region 5, homes had the lowest SDI value of any of the regions in 2018, but, in 2019, the SDI value for region 5 homes increased nearly to levels and then found in homes in regions 1, 2, and 4, although average values between sectors were non-significant (Fig. 2).

**Fig. 3:**
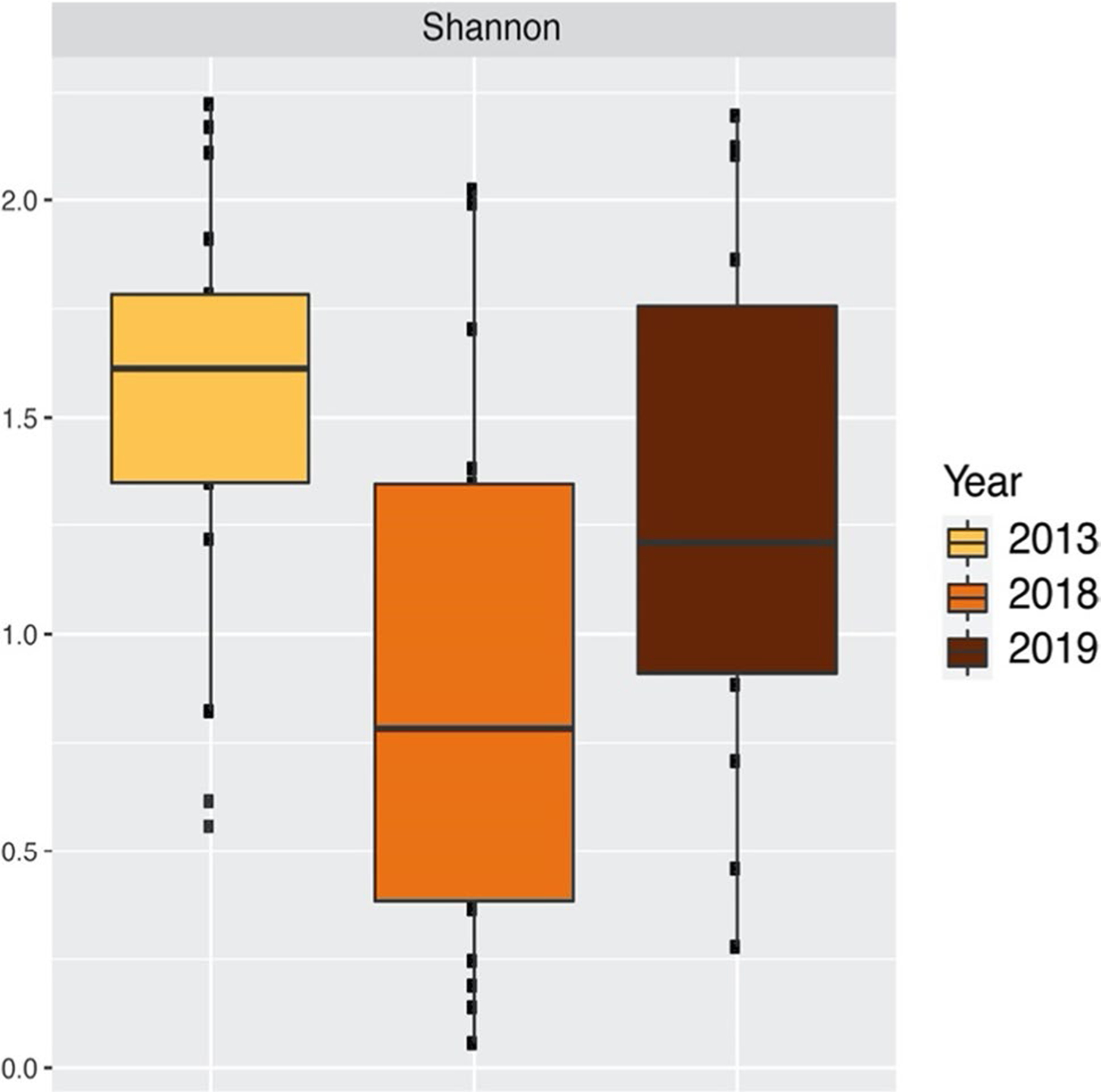
Shannon Diversity Index (SDI) values for the homes in eastern Puerto Rico sampled in 2013 and the average of the five regions in Piñones in 2018 and in 2019. A Tukey multiple comparisons of means test was applied to the SDI mean values and showed that there were significant differences between 2013 and 2018 (*p*-adjusted = 0.004)

Because we do not have a previous set of measurements either of this sector and/or of the participating households, we compared our results to a previous study of households located on eastern Puerto Rico (Bolaños-Rosero et al. 2013) in which also mold levels were assessed (Fig. 3). We found that the average SDI value in the Piñones community was significantly lower in 2018 than the SDI value in the eastern Puerto Rico homes in 2013 (*p*-adjusted value = 0.004) (Fig. 3). But, by 2019, the average SDI value in the Piñones community was near the average SDI value measured in eastern Puerto Rico homes in 2013 (*p*-adjusted value = 0.401) except for those households that have reported damage and little or no remediation (Fig. 3). The log of the average yearly outdoor mold-spore counts and their standard deviations (SD) in the greater San Juan area of Puerto Rico in 2017, 2018, and 2019 showed significant higher levels of outdoor mold spore counts in 2018 a year after Hurricane Maria (Table 3).

## Discussion

Homes in regions 1, 2, and 4 of the in Piñones community were generally spared major water damage. Therefore, the SDI values for the homes in these regions were little changed between 2018 and 2019. However, wind and heavy rain caused a tree to fall through the roof of one home in region 5, resulting in low SDI values. The other home in region 5, which was next door and occupied by the same extended family of people and dogs, resulted in cross-contamination between the homes. Fortunately, these homes were made of cement or vinyl. Once the roof was repaired and the furnishings removed or cleaned, the SDI value returned to a level comparable to regions 1, 2, and 4.

One of the homes in region 3 had obvious water damage, but both homes in region 3 were made of wood. As opposed to cement or vinyl, wood is much more susceptible to mold growth when it gets wet, and more difficult to dry-out. The SDI value for these region 3 homes did not improve between 2018 and 2019. This finding suggests that wood homes should be targeted for rapid repair and remediation in the wake of hurricanes. For these reasons, Ma and Smith (2020) suggested that “Disaster preparedness policies should raise structural standards for low-income housing to reduce risks of severe damage.”

In addition to impacts on housing, Hurricane Maria severely damaged much of Puerto Rico’s vegetation. Scholl et al. (2021) reported major defoliation due to Hurricane Maria. Hall et al. (2020) utilized field and remote sensing data to determine that 23% of island’s pre-hurricane forest had been destroyed. The category 4 Hurricane Maria killed twice as many trees as the category 3 Hurricane Hugo in 1989, and Maria broke 2- to 12-fold more stems than Hugo or George in 1998, also a category 3 hurricane (Uriarte et al. 2019). These major impacts on the vegetation appear to have released many phylloplane mold spores, i.e., molds that live on leaves, into the air, which led to the high outdoor mold counts in 2018 in San Juan AAAAI located 20.1 km from Piñones under the 30-km region reasonable estimation established (Katelaris et al. 2004) area (Fig. 3). However, by 2019, the outdoor mold-spore counts had returned to their pre-hurricane levels (Table 3).

*Cladosporium cladosporioides* and *Alternaria alternata* are well-known phylloplane molds (Miller 1992; Nix-Stohr et al. 2008; Hedayati et al. 2009). The increased level of these two molds was observed indoors in 2019 in the qPCR data. A group 2 mold like *Alternaria alternata* is an important source of allergens and may have implications for asthma prevalence (Grewling et al. 2020).

Although we do not have post-hurricane estimates of asthma in Piñones, the islands of St. Thomas, St. John, and the US Virgin Islands reported that there was a significant increase in patients being seen for diabetes-related and respiratory complaints, especially asthma (Chowdhury et al. 2019). It may take years to determine the impact of Hurricane Maria on asthma prevalence in Piñones.

Respiratory illnesses like asthma are expected to increase because of increases in exposure to pollen and mold resulting from climate change (D’Amato et al. 2020). Previously, urbanization was shown to be associated with reduced microbial outdoor exposure and increased contact with housing materials, which led to increased house and skin-mold diversity (McCall et al. 2020). It appears that climatic and urbanization changes may alter the prevalence of diseases associated with mold exposures.

There are many limitations to our study, including the low number of homes sampled. Some of the problems with recruitment arose because of the earthquakes that disrupted our recruiting efforts. In addition, some families did not allow the resampling of their homes in 2019. We also quantified a set of 36 molds as defined by ERMI under the assumption that the presence of these molds might help to assess the relationship between the presence of these molds, exposure, and possible impact on the respiratory health of these household inhabitants (Vesper & Wymer 2016).

However, and despite these limitations, our findings point out to the role that both house repairment and physical location might play in prolonged mold impacts after the exposure to severe weather events like hurricanes. Prompt action to repair major water damage seems to be relevant for quickly returning homes to typical mold levels in these households, and thus be a crucial factor for the short term improvement of indoor air quality after severe water exposure.

## Conclusion

After major atmospheric events such as hurricanes, fungal populations inside of the affected homes can have serious impacts into its microbial communities. Our results point out to the role that both location of the households and timing for damage repair play in the effects of indoor fungi after a hurricane. Additionally, the endurance of that effect through time and the possible long-term impact on the health of their inhabitants are suggested.

## Supplementary Material

SI

## Figures and Tables

**Table 1:** Characteristics of homes studied in the five regions of the Piñones community, shown as number of homes with the characteristic per total number of homes in that region. *AC* air-conditioning

Region	Water damage	Material	Ventilation	Dog
1	0/5	5 Cement	5 Natural	0/5
2	2/5	4 Cement; 1 Wood	5 Natural	1/5
3	1/2	2 Wood	2 Natural	0/2
4	0/5	5 Cement	4 Natural; 1 full AC	2/5
5	1/2	1 Cement; 1 vinyl	2 Natural	2/2

**Table 2:** Average (AVG) concentration in cell equivalents per mg dust (CE/mg dust) of each of the 36 Environmental Relative Moldiness Index (ERMI) molds in dust samples collected in 2018 and 2019 in Piñones homes compared using the Wilcoxon rank sum test (*p*-value), corrected for multiple comparisons using the Holms–Bonferroni test. Average sum of the logs of the group 1 or group 2 molds or ERMI values compared using the Student’s *t*-test (*p*-value). (*NM* no meaning; significant differences are bolded.)

Year	2018 AVG	2019 AVG	*p*-value
Group 1 molds	12	29	0.136
*Aspergillus flavus*			
*Aspergillus fumigatus*	2	3	0.211
*Aspergillus niger*	156	357	0.008
*Aspergillus ochraceus*	2	13	0.017
*Aspergillus penicillioides*	42,363	22,068	0.459
*Aspergillus restrictus*	94	31	0.095
*Aspergillus sclerotiorum*	68	35	0.479
*Aspergillus sydowii*	322	326	0.981
*Aspergillus unguis*	13	19	0.528
*Aspergillus versicolor*	668	164	0.384
*Aureobasidium pullulans*	170	87	0.321
*Chaetomium globosum*	73	111	0.534
*Cladosporium sphaerospermum*	10,082	468	0.388
*Eurotium amstelodami*	178	300	0.391
*Paecilomyces variotii*	16	14	0.882
*Penicillium brevicompactum*	3	12	0.064
*Penicillium corylophilum*	1	1	NM
*Penicillium crustosum*	7	615	0.247
*Penicillium purpurogenum*	2	9	0.073
*Penicillium spinulosum*	1	1	NM
*Penicillium variabile*	30	12	0.347
*Scopulariopsis brevicaulis*	4	8	0.292
*Scopulariopsis chartarum*	91	32	0.443
*Stachybotrys chartarum*	2	2	0.410
*Trichoderma viride*	18	9	0.416
*Wallemia sebi*	2318	2346	0.992
Sum logs group 1	28.9	30.7	0.45
Group 2 molds			
*Acremonium strictum*	1	3	0.032
*Alternaria alternata*	5	17	**< 0.001**
*Aspergillus ustus*	3	5	0.169
*Cladosporium cladosporioides* 1	508	1530	**≤ 0.001**
*Cladosporium cladosporioides* 2	3	3	0.928
*Cladosporium herbarum*	1	2	0.076
*Epicoccum nigrum*	1	1	0.254
*Mucor* group	14	50	0.184
*Penicillium chrysogenum* 2	1	1	0.324
*Rhizopus stolonifer*	4	41	0.068
Sum logs group 2	**4.8**	**7.8**	**< 0.01**
ERMI	24.2	22.9	0.52

**Table 3:** Log of the average yearly outdoor mold-spore counts and their standard deviations (SD) in the greater San Juan area of Puerto Rico in 2017, 2018, and 2019. Averages that are followed by the same letter are not significantly different

Year	Average	SD	Tukey
2017	4.6812	0.1417	**a**
2018	4.8593	0.1781	**b**
2019	4.7335	0.0895	**a**

## Data Availability

Data is made publicly available through the EPA website “SciHUB.”

## References

[R1] Blatter J, Forno E, Brehm J, Acosta-Pérez E, Alvarez M, Colón-Semidey A, Thorne PS, Metwali N, Canino G, Celedón JC (2014) Fungal exposure, atopy, and asthma exacerbations in Puerto Rican children. Ann Am Thorac Soc 11(6):925–932 24915164 10.1513/AnnalsATS.201402-077OC PMC4213992

[R2] Bolaños-Rosero B, Betancourt D, Dean T, Vesper S (2013) Pilot study of mold populations inside and outside of Puerto Rican residences. Aerobiol 29:537–543

[R3] Center for Disease Control and Prevention (2012) Asthma Surveillance- United States, 2006–2018.Morb Mortal Wkly Rep https://www.cdc.gov/mmwr/volumes/70/ss/pdfs/ss7005a1-H.pdf

[R4] Chowdhury MAB, Fiore AJ, Cohen SA, Wheatley C, Wheatley B, Balakrishnan MP, Chami M, Scieszka L, Drabin M, Roberts KA, Toben AC, Tyndall JA, Grattan LM, Morris JG Jr (2019) Health impact of Hurricanes Irma and Maria on St Thomas and St John, US Virgin Islands, 2017–2018. Am J Public Health 109(12):1725–1732 31622150 10.2105/AJPH.2019.305310 PMC6836793

[R5] Cowan KN, Qin X, Serrano KR, Sircar K, Pennington AF (2020) Uncontrolled asthma and household environmental exposures in Puerto Rico. J Asthma 21:1–11 10.1080/02770903.2020.1858861 PMC8215078 33272056

[R6] Cox J, Indugula R, Vesper S, Zhu Z, Jandarov R, Reponen T (2017) Comparative assessment of indoor air sampling and dust collection methods for fungal exposure assessment using quantitative PCR. Environ Sci Process Impacts 19(10):1312–1319 28858343 10.1039/c7em00257b PMC5884110

[R7] D’Amato G, Chong-Neto HJ, Monge-Ortega OP, Vitale C, Ansotegui I, Rosario N, Haahtela T, Galan C, Pawankar R, Murrieta-Aguttes M, Cecchi L, Bergmann C, Ridolo E, Ramon G, Gonzalez-Diaz S, D’Amato M, Annesi-Maesano I (2020) The effects of climate change on respiratory allergy and asthma induced by pollen and mold allergens. Allergy 75(9):2219–2228 32589303 10.1111/all.14476

[R8] El-Sayed A, Kamel M (2020) Climatic changes and their role in emergence and re-emergence of diseases. Environ Sci Pollut Res Int 27(18):22336–22352 32347486 10.1007/s11356-020-08896-w PMC7187803

[R9] García-Rivera EJ, Pacheco P, Colón M, Mays MH, Rivera M, Munet-Díaz V, González MDR, Rodríguez M, Rodríguez R, Morales A (2017) Building bridges to address health disparities in Puerto Rico: the “Salud para Piñones” project. Puerto Rico Health Sci J 36(2):92–100 PMC5645062 28622406

[R10] Grewling Ł, Bogawski P, Szymańska A, Nowak M, Kostecki Ł, Smith M (2020) Particle size distribution of the major Alternaria alternata allergen, Alt a 1, derived from airborne spores and subspore fragments. Fungal Biol 124(3–4):219–227 32220382 10.1016/j.funbio.2020.02.005

[R11] Hall J, Muscarella R, Quebbeman A, Arellano G, Thompson J, Zimmerman JK, Uriarte M (2020) Hurricane-induced rainfall is a stronger predictor of tropical forest damage in Puerto Rico than maximum wind speeds. Sci Rep 10(1):4318 32152355 10.1038/s41598-020-61164-2 PMC7062726

[R12] Haugland RH and Vesper SJ (2002) Identification and quantification of specific fungi and bacteria. US Patent No. 6,387,652, US Patent and Trademark Office, Washington, DC

[R13] Haugland RA, Varma M, Wymer LJ, Vesper SJ (2004) Quantitative PCR of selected Aspergillus, Penicillium and Paecilomyces species. Syst Appl Microbiol 27:198–210 15046309 10.1078/072320204322881826

[R14] Hedayati MT, Arabzadehmoghadam A, Hajheydari Z (2009) Specific IgE against Alternaria alternata in atopic dermatitis and asthma patients. Eur Rev Med Pharmacol Sci 13(3):187–191 19673169

[R15] Institute of Medicine of the National Academies (2004) Exposure assessment. In: Committee on damp indoor spaces and health The National Academies Press, Washington 25009878

[R16] Kassambara A (2018) ggpubr: ‘ggplot2’ based publication ready plots. R package version 0.2.3.999 Retrieved on November 28, 2021 from: https://cran.r-project.org/web/packages/ggpubr/index.html

[R17] Katelaris CH, Burke TV, Byth K (2004) Spatial variability in the pollen count in Sydney, Australia: can one sampling site accurately reflect the pollen count for the region? Ann Allergy Asthma Immunol 93:131–136 15328671 10.1016/S1081-1206(10)61464-0

[R18] Lewis LM, Mirabelli MC, Beavers SF, Kennedy CM, Shriber J, Stearns D, Morales- González JJ, Santiago MS, Félix IM, Ruiz-Serrano K, Dirlikov E, Lozier MJ, Sircar K, Flanders WD, Rivera-García B, Irizarry-Ramos J, Bolaños-Rosero B (2020) Characterizing environmental asthma triggers and healthcare use patterns in Puerto Rico. J Asthma 57(8):886–897 31187658 10.1080/02770903.2019.1612907 PMC8225466

[R19] Loyo-Berríos NI, Orengo JC, Serrano-Rodríguez RA (2006) Childhood asthma prevalence in northern Puerto Rico, the Rio Grande, and Loíza experience. J Asthma 43(8):619–624 17050228 10.1080/02770900600878693

[R20] Ma C, Smith C (2020) Vulnerability of renters and low-income households to storm damage: evidence from Hurricane Maria in Puerto Rico. Am J Public Health 110(2):196–202 31855476 10.2105/AJPH.2019.305438 PMC6951386

[R21] McCall LI, Callewaert C, Zhu Q, Song SJ, Bouslimani A, Minich JJ, Ernst M, Ruiz-Calderon JF, Cavallin H, Pereira HS, Novoselac A, Hernandez J, Rios R, Branch OH, Blaser MJ, Paulino LC, Dorrestein PC, Knight R, Dominguez-Bello MG (2020) Home chemical and microbial transitions across urbanization. Nat Microbiol 5(1):108–115 31686026 10.1038/s41564-019-0593-4 PMC7895447

[R22] McMurdie PJ, Holmes S (2013) phyloseq: an R package for reproducible interactive analysis and graphics of microbiome census data. PLoS ONE 8:e61217 23630581 10.1371/journal.pone.0061217 PMC3632530

[R23] Miller JD (1992) Fungi as contaminants in indoor air. Atmos Environ 26A:2163–2172

[R24] Nix-Stohr S, Moshe R, Dighton J (2008) Effects of propagule density and survival strategies on establishment and growth: further investigations in the phylloplane fungal model system. Microb Ecol 55:38–44 17436116 10.1007/s00248-007-9248-8

[R25] Pérez-Perdomo R, Pérez-Cardona C, Disdier-Flores O, Cintrón Y (2003) Prevalence and correlates of asthma in the Puerto Rican population: behavioral risk factor surveillance system, 2000. J Asthma 40(5):465–474 14529096 10.1081/jas-120018713

[R26] Quansah R, Jaakkola MS, Hugg TT, Heikkinen SA, Jaakkola JJ (2012) Residential dampness and molds and the risk of developing asthma: a systematic review and meta-analysis. PLoS ONE 7:e47526. 10.1371/journal.pone.0047526 23144822 PMC3492391

[R27] Scholl MA, Bassiouni M, and Torres-Sánchez AJ (2021) Drought stress and hurricane defoliation influence mountain clouds and moisture recycling in a tropical forest. Proc Natl AcadSci USA 118(7):e2021646118 10.1073/pnas.2021646118 PMC7896295 33563756

[R28] Shannon CE, Weaver W (1963) The Mathematical Theory of Communication University of Illinois Press

[R29] Sharpe RA, Bearman N, Thornton CR, Husk K, Osborne NJ (2015) Indoor fungal diversity and asthma: a meta-analysis and systematic review of risk factors. J Allergy Clin Immunol 135:110–122 25159468 10.1016/j.jaci.2014.07.002

[R30] Uriarte M, Thompson J, Zimmerman JK (2019) Hurricane Maria tripled stem breaks and doubled tree mortality relative to other major storms. Nat Commun 10(1):1362 30911008 10.1038/s41467-019-09319-2 PMC6433954

[R31] United States Census Bureau (2010) Summary File 1. 2010 Retrieved July 26, 2018 from https://www.census.gov

[R32] Vesper S (2011) Traditional mould analysis compared to a DNA-based method of mould analysis. Crit Rev Microbiol 37:15–24 20874612 10.3109/1040841X.2010.506177

[R33] Vesper SJ, McKinstry C, Haugland RA, Wymer L, Ashley P, Cox D, DeWalt G, Friedman W (2007) Development of an environmental relative moldiness index for homes in the U.S. J Occup Environ Med 49:829–833 17693779 10.1097/JOM.0b013e3181255e98

[R34] Vesper S, Wymer L (2016) The relationship between Environmental Relative Moldiness Index values and asthma. Int J Hyg Environ Health 219:233–238 26861576 10.1016/j.ijheh.2016.01.006

[R35] Wickham H (2009) ggplot2: Elegant Graphics for Data Analysis Springer-Verlag, New York

[R36] World Health Organization (2009) WHO guidelines for indoor air quality: dampness and mould World Health Organization, Europe 23785740

