# Supplementary material for: Impact of Hurricane Maria on mold levels in the homes of Piñones, Puerto Rico
Source: Air Qual Atmos Health. Author manuscript; Available in PMC 2023 Dec 26. (PMC10259649; doi:10.1007/s11869-022-01297-7)
Supplement: SI [file NIHMS1889077-supplement-SI.rtf]

shannon pairwise 2019Region    diff       lwr      upr     p adjS2-S1  0.22290654 -1.380847 1.826660 0.9895872S3-S1 -0.18104197 -2.145231 1.783147 0.9978311S4-S1  0.13170861 -1.600543 1.863960 0.9989765S5-S1 -0.29111520 -2.255304 1.673073 0.9867755S3-S2 -0.40394851 -2.368137 1.560240 0.9571222S4-S2 -0.09119793 -1.823450 1.641054 0.9997597S5-S2 -0.51402174 -2.478210 1.450167 0.9046195S4-S3  0.31275058 -1.757686 2.383187 0.9858123S5-S3 -0.11007323 -2.378123 2.157976 0.9998260S5-S4 -0.42282381 -2.493260 1.647613 0.9581493shannon pairwise 2018Region    diff       lwr      upr     p adjS2-S1 -0.36700205 -1.5821352 0.8481311 0.8717396S3-S1  0.62325545 -0.9454747 2.1919856 0.7234972S4-S1 -0.07892772 -1.2940609 1.1362054 0.9995541S5-S1 -0.94893093 -2.5176611 0.6197992 0.3618936S3-S2  0.99025750 -0.5252798 2.5057948 0.2939777S4-S2  0.28807433 -0.8575642 1.4337128 0.9285023S5-S2 -0.58192888 -2.0974662 0.9336084 0.7466107S4-S3 -0.70218317 -2.2177205 0.8133541 0.6038809S5-S3 -1.57218638 -3.3835999 0.2392271 0.1024208S5-S4 -0.87000321 -2.3855405 0.6455341 0.4098241
